# GPS-SLAM: An Augmentation of the ORB-SLAM Algorithm

**DOI:** 10.3390/s19224973

**Published:** 2019-11-15

**Authors:** Dániel Kiss-Illés, Cristina Barrado, Esther Salamí

**Affiliations:** 1 Karlsruhe Institute of Technology (KIT—Karlsruher Institut für Technologie), 76131 Karlsruhe, Germany; 2 Polytechnic University of Catalonia (UPC—Universitat Politécnica de Catalunya), 08034 Barcelona, Spain; cristina.barrado@upc.edu (C.B.); esalami@ac.upc.edu (E.S.)

**Keywords:** SLAM, GPS data, inertial, UAV, scarce dataset

## Abstract

This work presents Global Positioning System-Simultaneous Localization and Mapping (GPS-SLAM), an augmented version of Oriented FAST (Features from accelerated segment test) and Rotated BRIEF (Binary Robust Independent Elementary Features) feature detector (ORB)-SLAM using GPS and inertial data to make the algorithm capable of dealing with low frame rate datasets. In general, SLAM systems are successful in case of datasets with a high frame rate. This work was motivated by a scarce dataset where ORB-SLAM often loses track because of the lack of continuity. The main work includes the determination of the next frame’s pose based on the GPS and inertial data. The results show that this additional information makes the algorithm more robust. As many large, outdoor unmanned aerial vehicle (UAV) flights save the GPS and inertial measurement unit (IMU) data of the capturing of images, this program gives an option to use the SLAM algorithm successfully even if the dataset has a low frame rate.

## 1. Introduction

There are a lot of algorithms using the basic idea of Simultaneous Localization and Mapping (SLAM) [1,2,3]. In these methods, both the localization and the map creation are made by the algorithm itself. No more information than the images themselves is used. In many cases, however, there is additional information which was taken during the capture of the images. These can be the Global Navigation Satellite System (GNSS) data about the position of the camera and the inertial measurement unit (IMU) data for the orientation of the camera. The modified algorithm got the name GPS-SLAM since the most famous system of GNSS is the Global Positioning System (GPS). The abbreviation GPS is used throughout this paper every time when we refer to the data of the Global Navigation Satellite System.

ORB-SLAM is a very well-functioning SLAM algorithm written by Raúl Mur-Artal et al. [1,2]. It uses Oriented FAST (Features from accelerated segment test) and Rotated BRIEF (Binary Robust Independent Elementary Features) feature detector (ORB) presented by Ethan Rublee et al. [4]. We chose ORB-SLAM for this work because it is considered as “the most complete feature-based monocular visual SLAM system” [5]. With ORB-SLAM, one can create the map and the trajectory of the camera positions successfully for many datasets. However in some cases, these algorithms fail too. They can lose track and recover only much later. In datasets with low frame rate, consecutive images do not have enough coverage and tracking is lost due to the lack of continuity. Also, frames can be detected of false orientation, position, or even both. The algorithm may not recognize that the agent (robot, drone, etc.) is at the same spot if the images were taken of different directions. The scene has different features from different viewpoints and because of the accumulated drift the algorithm may localize itself incorrectly.

Some of these failures can be solved: the loop closure part helps to correct the accumulated drift. However, this does not happen in every occasion since there can be datasets where no loops are made. In other cases, the loop is simply not detected because the images were taken from opposite directions and so the features are different.

The SLAM algorithm ORB-SLAM [1,2] was tested on datasets which have high frame rates. In the majority of cases the difference between two images of the dataset is low. They often have 10 images in every second, so even if the dataset was captured with rapid changes, two consecutive images have a lot of correspondences and similarities.

In other situations datasets can be more scarce. Many unmanned aerial vehicle (UAV) flights [6] take images only every 1 or 2 s. They process the data in other ways for which this frame rate is sufficient. For example, the Structure from motion (SfM) method is a post-processing technique which does not need datasets with high frame rates. But for SLAM algorithms, a scarce dataset is harder to track.

In this work we present GPS-SLAM, an improvement of ORB-SLAM by using GPS and IMU data of the images to make it more robust for datasets with low frame rate. The work was urged by a database where ORB-SLAM failed to keep track. The SLAM algorithm was tested with different configurations to make the tracking more robust. However, these lead only partly to a better tracking. We use GPS and inertial data to determine the next frame’s position. They are adjusted and optimized later on by the parts of the original algorithm.

## 2. Materials and Methods

In this section the modifications of the ORB-SLAM algorithm will be discussed, which methods and functions were changed, and how the augmented algorithm works. First, the original algorithm will be described briefly. After that, in order to reason why these changes were made, some parts of the results will be anticipated, without going into the details. In this work we generally speak about ORB-SLAM, but it should be mentioned that the augmented algorithm is the modification of ORB-SLAM2 [2]. The changed parts of the code are to find in the Appendix A.1, Appendix A.2 and Appendix A.3.

### 2.1. Brief Description of ORB-SLAM

ORB-SLAM uses a set of consecutive images to make a map and localize itself in it. This can happen real-time, as the agent processes every image as soon as they are made, or afterwards from a dataset. From every image—the so-called frame—important visual points (features) are extracted. By these features, the images can be compared to each other and an initial map is created based on the movement between the frames. The map consists of map points which are the 3D-projections of selected features, and frames which are the positions where the correspondent images were made. The 3D-projection is possible since the parameters of the camera are available.

After a successful initialization with each new frame the new position is tracked and the map is updated by new map points. If necessary, redundant and/or erroneous frames and map points are deleted. At the end the trajectory of the tracking is available, and the map points can also be retrieved.

### 2.2. SLAM Algorithms and the GPS Data

SLAM algorithms were invented in order to create a map about the surroundings of an agent and that this agent can localize itself in this map. Survey [7] classifies SLAM in two types: Filtering based (i.e., FastSLAM [8] and OrbitalSlam [9]) and Analysis based (i.e., PTAM [10] and LSD-SLAM [11]). Both types of SLAM use no more information than the images and the Visual Odometry fundamentals [12].

Since SLAM algorithms are capable of the previously mentioned task, at first glance the use of the GPS data can seem to be redundant. However, neither the positions by the SLAM algorithm nor the GPS data are flawless. Previous works [1,2] prove that the SLAM algorithm can accumulate a lot of drift, and that this drift is not always corrected. Regarding the GPS data, there can always be errors in the measurement of the position which makes the GPS data slightly imprecise as well. SLAM can correct the imprecision of the GPS data by the bundle adjustment and its optimization methods. The goal of proposed GPS-SLAM is to exploit the benefits and advantages of both systems and get results of better quality by combining them.

A reversal approach is proposed by Kleinert et al. [13]. A vehicle navigates using a classical Kalman filter, fed with data from the inertial unit and the raw GPS pseudo-ranges. In order to solve the problem of navigation in GPS-denied scenarios, the localization is augmented with the coordinates of landmarks obtained with SLAM.

SLAM has mainly been deployed in ground vehicles and robots, but recently works start to apply SLAM also by UAV [11,14]. For instance, in [15] a swarm of UAVs works collaboratively to produce a map using a distributed version of monocular SLAM. Nowadays almost every UAV is mounted with GPS sensors, which are cheap and sufficiently precise for the navigation of drones. To use already available data (GPS) to augment an already existing real-time system (SLAM) seems to be a plausible solution in order to improve the results of the algorithm.

The main goal of the proposed augmentation of the original algorithm was to get a better quality 3D map in real time. With GPS data, the quality of the 3D reconstruction can be increased. There are other algorithms which can make a very good quality map using only the images of a dataset or additionally the GPS data, but they need much more time for the calculations [16,17]. Here comes the advantage of SLAM: the creation of the trajectory and map points are real-time and so the 3D reconstruction of the map can be made in a very short time after the flight or a general movement is finished.

### 2.3. The Modifications Leading to GPS-SLAM

**Figure 1:**
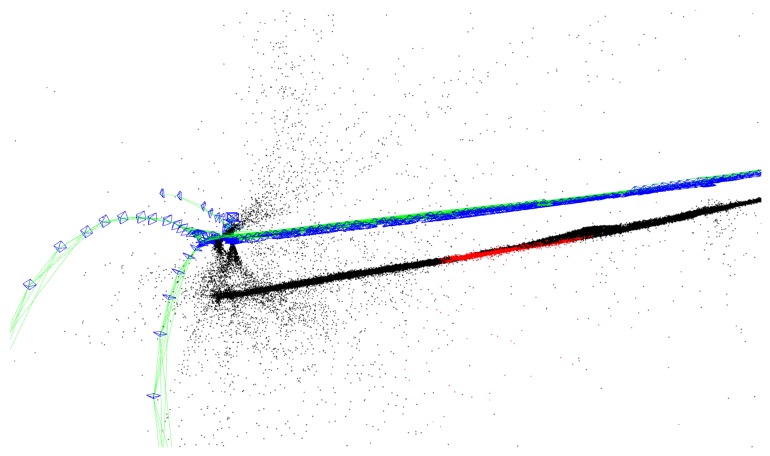
False frame and map point detection by Oriented FAST (Features from accelerated segment test) and Rotated BRIEF (Binary Robust Independent Elementary Features) feature detector-Simultaneous Localization and Mapping (ORB-SLAM).

ORB-SLAM worked well for a lot of datasets with the extraction of 2000 features from the images. In our case this number of features seemed not to be enough. The number of extracted features was increased to 6000. With this an unexpected situation happened. Instead of having a denser map and a more reliable localization, false frame and map point detection was observed (see in Figure 1).

#### 2.3.1. Using the GPS Data

As by increasing the number of extracted features we did not make much progress, we tried to use other data sources. Since the GPS and inertial data of the flight was available, it was plausible to use them to help the tracking. The GPS data can be used best in the Tracking part in which the position for the next frame is predicted. The original algorithm uses a velocity model (*TrackWithMotionModel*) based on the transformation matrix between the poses of the last two frames. This works well if the movement of the camera does not change but can fail in other cases. The velocity model was replaced, and the algorithm determined the next position based on the GPS and inertial data.

For this the corresponding GPS data was read in for every image, in the form of the XYZ coordinates (see Equation (1)) and angles from the IMU (yaw—α, pitch—β, roll—γ). The single rotational matrices (see Equation (2)) were calculated from these angles and then multiplied together to form the orientation matrix of the camera (see Equation (3)). From the rotational matrix and the position vector, the pose matrix (see Equation (4)) can be defined.

(1) $$pos=\;\left[\begin{array}{c} x\\ y\\ z \end{array}\right]$$

(2) $$R_{x}=\;\left[\begin{array}{ccc} 1 & 0 & 0\\ 0 & \cos\alpha & -\sin\alpha \\ 0 & \sin\alpha & \cos\alpha \end{array}\right]$$

(3) $$R_{total}=R_{z}*R_{y}*R_{x}$$

(4) $$T_{frame}=\left[\begin{array}{cc} \left[\begin{array}{cc} \; & \;\\ \; & R_{total}\\ \; & \; \end{array}\right] & \left[\begin{array}{c} pos \end{array}\right]\\ 0 & 1\; \end{array}\right]$$

The initialization process is not changed. The algorithm is left on its own to make an initial map and localize itself. There are 3 coordinate systems in the modified algorithm:

- SLAM world coordinate system (*fix*)—noted with *w*
- SLAM camera coordinate system (*changes*)—noted with *c*
- GPS coordinate system (*fix*)—noted with *GPS*

The two SLAM coordinate systems have the same scaling: they differ only in their orientation. The GPS coordinate system has both a different scaling and orientation in comparison to the two other coordinate systems of the SLAM. The SLAM world coordinate system has its origin where the first frame is. This frame never gets deleted from the system and this coordinate system does not change during the execution. The SLAM camera coordinate system is where the current frame is, and *changes* with every new frame. The current position and orientation are always expressed in the current SLAM camera coordinate system.

Since the SLAM camera coordinate system changes with every new frame, a rotation matrix cannot be determined which would describe the transformation from the GPS coordinate system to each SLAM camera coordinate system. Because of this we calculated the rotation matrix from the GPS to the SLAM world coordinate system. These coordinate systems are fixed, as is the rotational matrix.

To calculate the rotation matrix, the translation vector between the first and the initializing frame was used. This translation vector has its description in both of the fixed coordinate systems:

- SLAM world coordinate system—$t_{w}$ (see Equation (6))
- GPS coordinate system—$t_{GPS}$ (see Equation (5))

**Figure 2:**
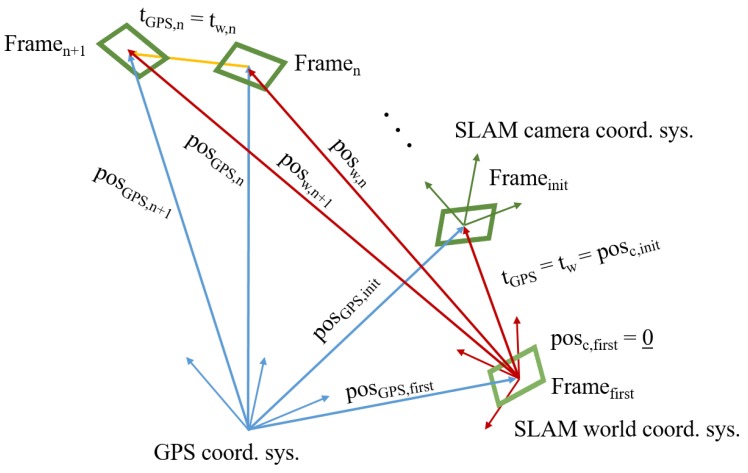
The coordinate systems and vectors of the transformations.

Figure 2 illustrates the 3 coordinates systems (GPS, SLAM world, SLAM camera), the position vectors, and the translation vectors based on which the rotation matrix is calculated between the GPS and the SLAM world coordinates systems.

Having the translation vector with its description in both coordinate systems, the rotation matrix can be calculated. The detailed calculations are found in Appendix A.1, as it is in the code. At last we have the rotation matrix between the GPS and SLAM world coordinate system $R_{GPSw}$. Any vector can now be transformed between the coordinate systems (see Equation (7)).

(5) $$t_{GPS}=pos_{GPS,init}-pos_{GPS,first}$$

(6) $$t_{w}=R_{cw}*\left(pos_{c,init}-pos_{c,first}\right)$$

(7) $$R_{GPSw}*t_{GPS}=t_{w}$$

The other part of the new data is the rotation between the frames. The rotation matrix of two consecutive frames is calculated as in the following equation (see Equation (8)).

(8) $$R_{n-1,n}=R_{n}*R_{n-1}^{-1}$$

This rotation matrix does not have to be transformed between coordinate systems, because it describes the change of the orientation relatively.

With each new frame, the difference in the pose (rotation and translation) of the new and the last frame is calculated in the GPS coordinate system (see in Appendix A.2). This is then transformed into coordinates of the SLAM world coordinate system. In this way, the GPS data can be used to determine the next position in the world of SLAM.

At last the transformation between the SLAM world and camera coordinate systems has to be made. This is possible since the last frame’s pose stores the transformation matrix between these coordinate systems. These transformations are described here briefly (see Equations (9) and (10)) and detailed in Appendix A.3.

(9) $$t_{c}=R_{wc}*R_{GPSw}*t_{GPS}$$

(10) $$pos_{c,new}=R_{n-1,n}*\left[pos_{c,last}+t_{c,new}\right]$$

The rotation part (orientation of the camera) is simply multiplied by the change of rotation between the new and the last frame described by the GPS data (see Equation (11)):

(11) $$R_{c,new}=R_{n-1,n}*R_{c,last}$$

After calculating the new orientation and position this pose matrix is given to the algorithm to use as the next pose. The following parts of the code were not changed, *matches* of the features are searched by projecting them into the same space based on the new position. Outliers are discarded and at the end the function returns whether the tracking was successful or not.

**Figure 3:**
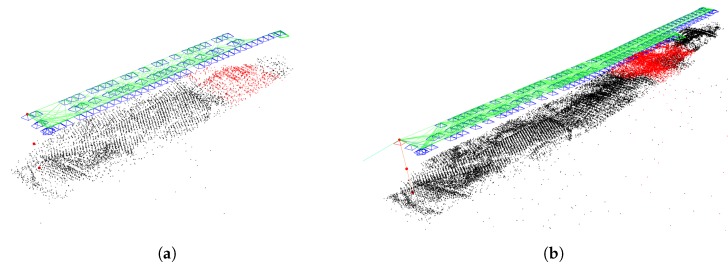
Differences between the original Figure 3a and the augmented version Figure 3b. (**a**) Original ORB-SLAM2. (**b**) Augmented GPS-SLAM.

The first experiences of applying the new method were positive: fewer false frames, denser map, the tracking was lost fewer times. In Figure 3 it can be seen that the original algorithm loses track in the middle of the lanes and has fewer map points, while the augmented version is able to track the entire lanes and the created map is denser. This will be discussed in detail later in Section 3.

#### 2.3.2. Modification of the “*TrackReferenceKeyFrame*” Method

**Figure 4:**
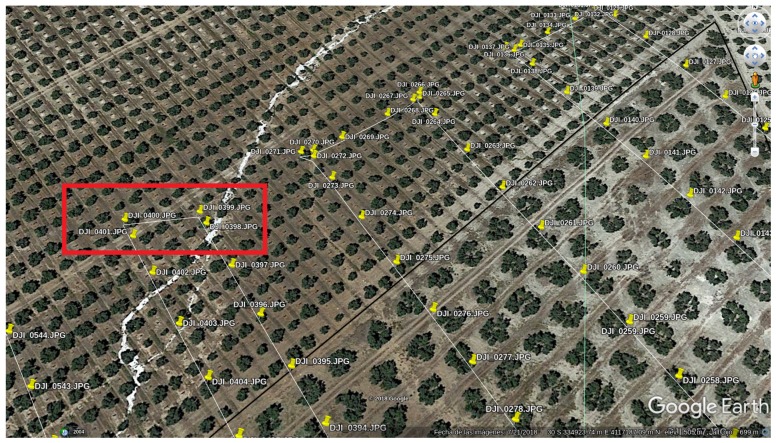
The turn with the insufficient number of images.

However, not all of the problems were solved with this modification. There is an image in the dataset where a turn of 180${}^{\circ }$ takes place (see in Figure 4) and at this point the tracking was lost every time. The algorithm changed to the *TrackReferenceKeyFrame* method, as the tracking by the *TrackWithGPSData* method was not successful. But this method could not bear with such a big turn either. In case of tracking by *TrackReferenceKeyFrame* the method optimizes and refines the position of the last frame to determine the next frame’s position. In case of small changes this approach is successful. Nonetheless, this turn in particular is very big. Using the last frame’s position is not accurate enough to refine the position and find the correct one.

Since we already calculated the new position before based on the GPS and inertial data, it was suitable to use it in case of tracking by reference keyframe (*TrackReferenceKeyFrame*) as well. Undoubtedly we saw that this new position did not lead to a successful tracking by *TrackWithGPSData*, but it still seemed to be a better guess than using the last position because the change is so big.

Consequently, the *TrackReferenceKeyFrame* method was modified so that it uses the position determined by the GPS and inertial data as a guess for the new true position. This turned out to be a good intuition, the mentioned turn of 180${}^{\circ }$ was successfully tracked from this point on in every test run.

#### 2.3.3. Relocalization Candidates Determined by the Position of the Frames

The next challenge was the improvement of the relocalization. At a certain frame, the tracking completely was lost every time in the testing phase. This was unexpected since the original, unchanged version could relocalize more often even with a lower number of tracked frames and more losses of tracking. The problem was not the available time. The time of execution was in the same order of magnitude the same as in the original version. This meant that the algorithm does not run out of time, but the adequate frames are not considered as candidates for the relocalization.

To make sure of this, the method was changed to look for candidate frames for the relocalization in the whole keyframe database. With this change the relocalization was successful again. But, as expected, searching for the candidate frames between every frame takes much more time. We arrived at times of the execution of 1.5 or even 2 s only for this single method. This meant the relocaliztion itself works, only the candidate frames are not determined in the right way. The source of it could be that candidates are searched for by looking at features that they share with the current *lost* image. This search takes place in the upper level of the scaling pyramid where features are simplified a lot. Since the images are very similar in this dataset, the comparison of the simplified images leads to false candidate detection.

Instead of choosing the candidate frames for finding the track again by looking at similar features, we looked at the positions of the frames. We looked for frames which are in a close neighbourhood to the current *lost* frame. Calculating the distances between the *lost* and every other frame proved to be very time consuming. Since the distance to the first frame is stored for every frame, based upon this the ones which are in a certain distance from the first frame are chosen as candidates. With this change, the relocalization became successful many more times and the execution times were again in the right order of magnitude.

### 2.4. The Dataset

For the evaluation of GPS-SLAM, the image dataset of [6] was used. The dataset consists of 774 consecutive images about an olive-tree field captured by a UAV. The flight took place at the same altitude during the whole time. The images were taken every 2 s from the same direction, and the camera was directed sheer to the ground. The original resolution of the photos was 4000 × 3000 pixels, but in order for the algorithm to deal with them, they were resized to 400 × 300 pixels.

The augmented version needs, beside the images, the GPS data as well. These are stored in a text file with each line representing one image. Every line contains the corresponding angles and coordinates.

## 3. Results and Discussion

### 3.1. The Experiment Design

ORB is based in the FAST feature matching algorithm, originally proposed by Rosten and Drummond [18]. As the authors explain in their paper, the “generalized Monte–Carlo based techniques (…) escape local optima easier by randomly perturbing the system”. Since the results are partially random, more than 1 execution was necessary to get real data from the algorithm. With 2 slightly different adjustments regarding the number of extracted features from an image, the 2 algorithms were run 10 times on the same olive-tree dataset [6]. The algorithm was executed on a desktop PC with an Intel Core TM i7-3770 (3.40 GHz) with 16 GB of memory and a NVIDIA GeForce GTX-650 graphical card.

### 3.2. Aspects of Comparison

During testing, for every run the same data were extracted. We checked the *Last Tracked Frame*, how many *times* the tracking was *Lost*, the *Number of Lost Frames*, and calculated the *Lost Frames per Losses* from the last two. Moreover, the *Number of Calls to TrackReferenceKeyFrame* and the *Number of Erroneous Frames* was counted as well. The algorithms track a different number of map points—depending on the number of extracted features—thus we counted the *Number of Map Points* as well. Since the GPS data is already used and a comparison between the results of the SLAM algorithm and the GPS data would not be a good measurement of accuracy, the *Percentage of False Map Points* was counted. It is known that by this dataset a piece of the earth’s surface was captured and so we can clearly detect false map points (“under the ground” or “in the air”). In addition, the *Mean*, *Standard Deviation*, and *Median of the Tracking Time*, as well as the *Mean of the Relocalization Time* was measured to check how fast the algorithm works.

The list of the aspects of comparison:

- Last Tracked Frame
- Lost (times)
- Number of Lost Frames
- Lost Frames per Losses
- Number of Calls to *TrackReferenceKeyFrame*
- Number of Erroneous Frames
- Number of Map Points
- Percentage of False Map Points
- Mean Tracking Time
- Standard Deviation of Tracking Time
- Median of Tracking Time
- Mean Relocalization Time

The aspect *Number of Frames tracked by TrackReferenceKeyFrame* was proposed to ascertain how successful the *TrackWithGPSData* method is which uses the GPS and inertial data for the determination of the next position. In this we counted the number of executions of the other method (*TrackReferenceKeyFrame*) responsible for the tracking: if the method *TrackWithGPSData* fails to locate the camera, the program jumps to another method *TrackReferenceKeyFrame*. By counting the times the other tracking method is executed, we can make sure how robust the principal method (*TrackWithGPSData*) of tracking is.

The *Number of Erroneous Frames* stands for the frames which are clearly falsely detected:

- a frame which is not in the plane of the UAV flight
- a frame with erroneous orientation

Since the images were taken more or less in a plane so that orientation of the camera was pointing at the ground, these frames are easy to detect.

*False Map Points* are detected by their *z* coordinate. When they “do not lie on the ground”, they are considered as false. It should be mentioned that the *Tracking Time* includes the process of relocalization since the latter is part of the former. When the *Mean Relocalization Time* increases, this makes the *Mean Tracking Time* higher, too. In general, *Tracking Time* is the time for processing one image from its read-in until detecting where the camera pose was and registering the new map points.

The *Mean of the Relocalization Time* does not stand for the time which goes by between the loss and recovery of the track but for the time of the execution of the *Relocalization* method. In this method the candidate frames are gathered, and from these the adequate frame for the relocalization is chosen.

### 3.3. Comparison of the Algorithms Using 2000 Features

First, in Table 1 the average values for 10 runs of each algorithm are shown, made with 2000 features. Some values can only be whole numbers for single runs, but we kept these average decimal numbers for the sake of the statistics. The column *Imp. (Improvement)* stands for whether the algorithm in the right column (GPS-SLAM in this case) has improved compared to the algorithm in the left column (ORB-SLAM in this case). Green tick stands for improvement while red cross means worse performance. The improvement in percentage is in the 4th column, while the last column presents the statistical significance.

The data in Table 1 can be divided into 2 parts: one group about the frames and map points and the other about execution times. Statistics regarding the frames gave mixed results: in some aspects the original was more successful, while in other points the augmented algorithm performs better. In this database of 774 images the GPS-SLAM can track the path longer, the *Last Tracked Frame* was always a latter frame than in the case of the original. It loses track more often, though. Even so, the augmented algorithm tracks much more frames (76.2 to 280 lost frames respectively). If we come to the case that the track is lost, GPS-SLAM recovers much faster than the original ORB-SLAM algorithm: the *Number of Lost Frames* is much higher in the case of the original algorithm, there are almost 4-times more lost frames in its executions. This means that although the number of how many times the tracking was *Lost* is higher in the case of the GPS-SLAM, if this happens the augmented algorithm recovers much faster, having a small number of *Lost Frames per Losses*.

By counting the times in which the method *TrackReferenceKeyFrame* was used, one can tell how robust the tracking with GPS data is. As we can see in the statistics, the GPS-SLAM had to use this other method fewer times, which means that the determination of the next pose based upon GPS data is more accurate than a prediction by a velocity model. Of course, this is helpful above all in turns and curves where a prediction by a velocity model cannot be true.

During the evaluation part, it could be observed that in some cases there are erroneous frames. They might not be in the plane in which the UAV performed the flight, or their orientation was falsely determined, not perpendicular to the ground. For this situation, another statistical point was invented to count the number of these erroneous frames. We can see in Table 3 that the two algorithms in general have a low number of erroneous frames (regarding that there are 774 images in the dataset), but we can declare that in this aspect the GPS-SLAM worked slightly better.

Changing to the second part of the statistical data, we can see the execution times of some chosen functions. We were interested in how long the tracking lasts and, in case of a relocalization, how much time it consumes. It can be seen that the original algorithm is in general faster than GPS-SLAM. The main reason of this is the relocalization process. Looking at those times, it can be stated that the augmented version takes more than 5 times longer executing this method than the original one. The execution time of this method is in close relation with the number of candidate frames. The candidates go through a matching process which can take a significant time, often between 0.1 and 0.2 s, if the number of candidates is higher (e.g., 40–80 candidates). We can see too that in general the relocalization takes longer than the tracking itself which the relocalization is part of. This means that without the need of relocalizing the tracking is fast, but if it is needed, the time for tracking increases.

This yields us an explanation, too, for why the original algorithm is less effective regarding the relocalization. It is known that the time-consuming part of this method is the matching of frames, and since the time of this function in the original version stays low, this means that there are only a few frames chosen as candidates and that these frames are not the correct ones. The augmented version, although slower, works better because between the candidates the correct frame can be found as well.

The *Mean Tracking Time* is under 0.1 s for both versions, but it has a bigger standard deviation in the case of the augmented one. This comes above all from the relocalization process as stated in the last paragraph. At this point we should mention that since the tracking time can go above 0.1 s (in case of trying to find the track), the augmented version would not be suitable in this form for real-time executions for datasets with higher frame rate (e.g., 10). The necessary time would be longer than what is availabe for the algorithm. On the other hand, with some adjustments it can be made suitable for datasets with a higher frame rate by only considering a limited number of candidates and so limiting the time which the method can consume. However, it does not play an important role for our and other scarce databases because consecutive images are coming only every 1 or 2 s. Although the processing times are higher than those in the original version, they stay in the same order of magnitude. This means that with the given frequency (0.5 or 1 fps), the algorithm remains real-time without any question.

To summarize the results of the first executions with 2000 features, we experienced improvements in some aspects while got worse results in other points. The tracking got more stable in the sense that it goes farther, if the track is lost the relocalization is much faster, the track is lost only for a couple of frames on average, and we have fewer erroneous frames. Moreover, the new method *TrackWithGPSData* is robust, as the other tracking method *TrackReferenceKeyFrame* does not have to be used as many times as in the original version. On the other hand, the track is lost more times, there are more falsely detected map points, and the time of tracking became greater in general.

In the first phases of the development of the augmented version we already experimented with the number of extracted features for both algorithms. Since interesting and important results were found, they will be presented now, in which the algorithms were run with 6000 extracted features.

### 3.4. Comparison of the Algorithms Using 6000 Features

At the beginning, the original algorithm was tested with different numbers of extracted features. We experienced different results for lower (2000) and higher (6000) numbers of features, which is why later both algorithms were tested and compared in both settings.

In Table 2 the results for the second part with 6000 features are shown. Again, both algorithms were run 10 times and the results were averaged.

This time, the GPS version clearly outperforms the original one. In all aspects regarding the frames and map points it produced better results. The tracking goes farther, the number of losses of track is reduced, there are fewer lost frames, relocalization happens faster, and there are almost no erroneous frames and map points. The augmented algorithm with 6000 features had to use the *TrackReferenceKeyFrame* method fewer times than with 2000 features.

Looking at the times, they have increased for both version since there are more features to extract and to work with. Again, the *Mean Tracking Time* is influenced by the relocalization, which takes longer in the augmented version, as described before. But these times are still allowed for scarce datasets.

We have seen that the augmented algorithm can deal much better with a higher number of features than the original version. It is clearly better than with 2000 features. In the case of the original version this is not true. Although the number of lost frames is lower, and some aspects seem to be slightly better as with 2000 features, since there are almost 7 times more erroneous frames and much more false map points as with 2000 features we cannot regard it better as the other one.

To have a clearer view we now compare the differences within the versions. We start with ORB-SLAM executions with 2000 and 6000 features.

### 3.5. Comparison of ORB-SLAM Using 2000 and 6000 Features

To find the better adjustment for both versions, we compared them separately. Table 3 details the results of the comparison of ORB-SLAM run with different numbers of extracted features.

**Figure 5:**
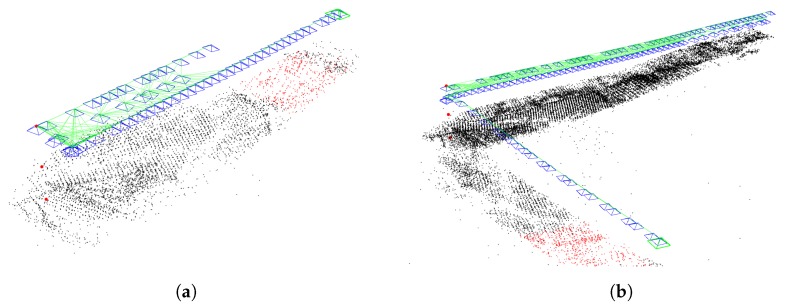
Significantly different outputs of ORB-SLAM with different numbers of extracted features. (**a**) ORB-SLAM with 2000 features. (**b**) ORB-SLAM with 6000 features.

It can be seen that contradictorily to the first thoughts, more features can be helpful for a tracking of better quality, while the original version works better with fewer features. If more points are extracted from the images it leads to false map point and false frame detection, which ultimately decreases the quality of tracking significantly. Only two aspects give better results for the first sight, but of these the *Last Tracked Frame* is only slightly better, while the *Number of Lost Frames* is not a good benchmark if there are so many erroneous frames, as in this case. We can say that the original ORB-SLAM algorithm, although not perfect, works better and more robust with fewer features than with more. In our point of view, the tracking should rather be lost but precise in general, than track longer but with false results. Figure 5 shows examples of the execution of ORB-SLAM with different numbers of extracted features.

Because of this, in the ultimate comparison we chose the setting of 2000 features for the original algorithm to be compared with the better one of the augmented version.

### 3.6. Comparison of the GPS-SLAM Using 2000 and 6000 Features

Next we looked at the data of the GPS-SLAM version, whether the quality of tracking has increased in this case.

**Figure 6:**
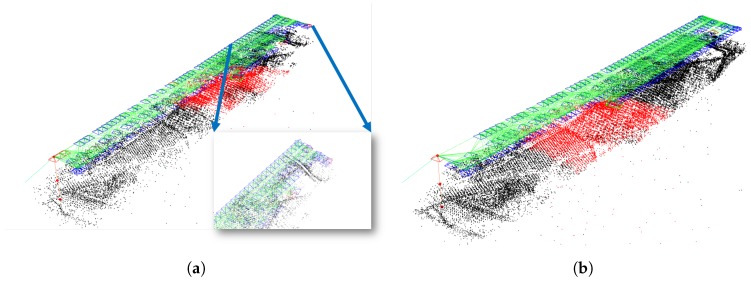
Slightly different outputs of GPS-SLAM with different numbers of extracted features. (**a**) GPS-SLAM with 2000 features. (**b**) GPS-SLAM with 6000 features.

As the results show in Table 4, for the augmented version a higher number of extracted features improves the quality of tracking. With the exception of one aspect, in which the difference is very low and not significant, the algorithm works much better with more features. Execution times as always remain greater since more features need more time to deal with. Figure 6 shows a pair of executions of GPS-SLAM with 2000 and 6000 features, respectively.

### 3.7. Comparison of the Best Settings of ORB-SLAM and GPS-SLAM

At last we compare the better settings for each version. As Table 5 shows, GPS-SLAM worked better than ORB-SLAM when comparing them in their best settings.

It can be stated that the augmented algorithm has better results regarding the tracking, but it also takes longer for processing an image. However, as the algorithm was developed for uses with scarce datasets where the frame rate is low and the time between 2 consecutive images is higher, this longer time of processing an image does not play a significant role. It remains under the available time for an image and so the algorithm remains real-time. In other aspects the tracking is of *much better quality*, mostly because it can track much more frames and its determination method is correct more times than the prediction method of the original algorithm.

Another advantage of GPS-SLAM is that since the determination of the next frame is better, the number of features to be extracted from an image can be higher, because this will not lead to an erroneous frame and map point detection. This way, with a higher number of features the generated map is denser, which is more suitable for 3D map creation.

**Figure 7:**
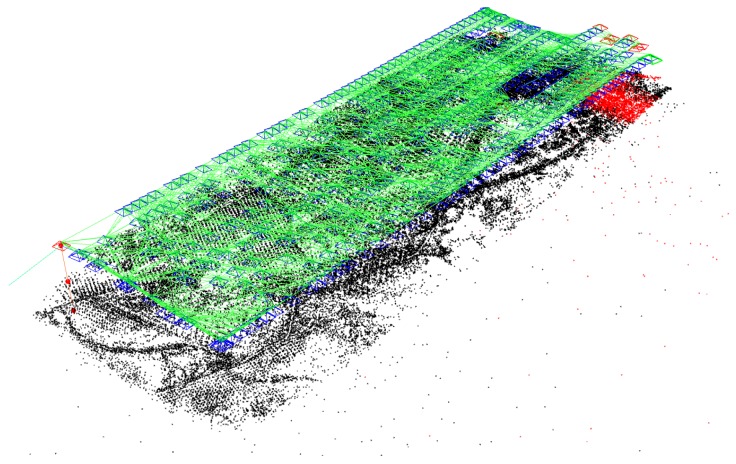
Execution until the last frame of GPS-SLAM with 6000 features.

In Figure 7 the final result of the best working algorithm is shown (GPS-SLAM 6000 features). We got a dense map with only a few false map points and frames.

### 3.8. Frame Rates—Fastest Execution with Good Results

Until this point, all results were made with executions of 0.5 fps. This was the original speed of the sequence, capturing the images every 2 s. However, we would like to know how fast the algorithm can become. During testing we also retrieved the maximum times of executing the *Track* and the *Relocalization* methods. Although the mean times of tracking and relocalization were always under 0.1 s, the maximum values tended to differ a lot between them and sometimes (on very rare occasions) get even higher than the available time. Every time the available time (2 s with 0.5 fps) is not enough, the algorithm starts immediately processing the next image when it finishes with the last one. In the normal case, when the processing time is less then the available, the algorithm waits with the next image until the available runs down. In the case of after processing, when the dataset already exists, this phenomenon is not important. The algorithm waits a bit longer and then processes the next image. In real time this would mean a small slip between making the images and processing them. However, since these salient values occur only a very few times, the algorithm very probably can catch up with the next image, if the processing time for the next image remains lower than the available time. This way, the algorithm can remain real-time despite the processing time sometimes exceeding the available time.

After making sure that although the processing time may go above the available time this does not crash the tracking, we would like to find out which is the highest frame rate which still yields a good quality tracking. We tried frame rates from the original 0.5 fps to 1, 2, and 4 fps. We used GPS-SLAM with 6000 features and the results are the averaged values of 3 executions. The same data was extracted for these configurations, and the most important aspects were compared to each other. In the following diagrams we show how the quality of tracking changes with the increment of the frame rate.

**Figure 8:**
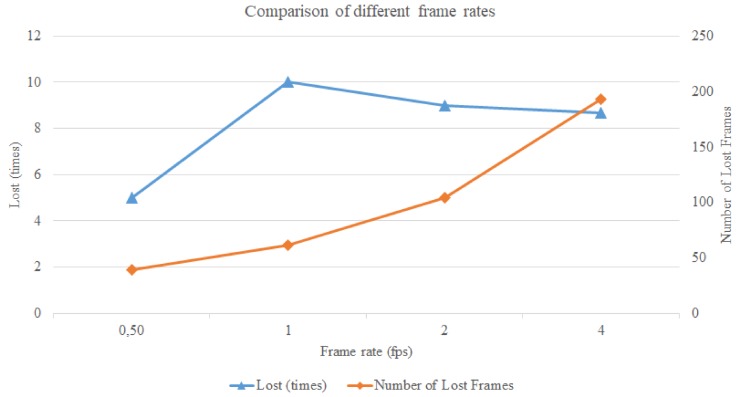
The influence of the frame rate on how many times the track gets *Lost* and on the *Number of Lost Frames*.

Figure 8 shows how the frame rate affects how many times the track was *Lost* and the *Number of Lost Frames*. With increasing frame rate the track is lost more times in general, and the *Number of Lost Frames* is higher as well. With a frame rate of 2 fps there are already twice as many *Lost Frames* as with the original frame rate and with 4 fps this number is even higher, around 4 times bigger than in the case of the original one.

**Figure 9:**
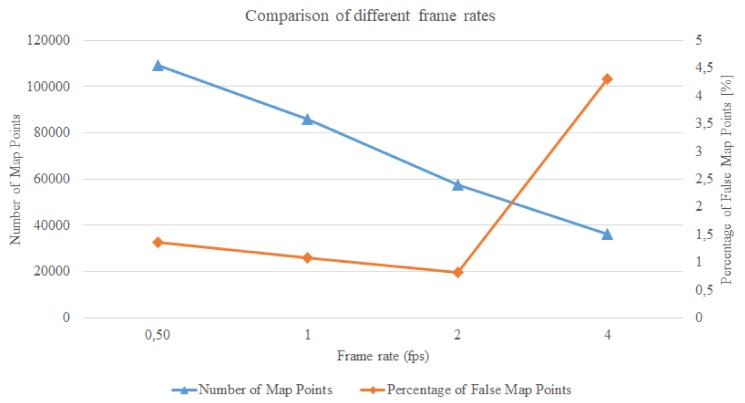
The influence of the frame rate on the *Number of Map Points* and the *Percentage of False Map Points*.

In Figure 9 we can see how the *Number of Map Points* and the *Percentage of False Map Points* change with respect to different frame rates. With increasing frame rate the *Number of Map Points* decrease continually. The *Percentage of False Map Points* does not change significantly until 2 fps, but then with 4 fps we can see a big rise in their number.

It can be stated that the best results come with the original, lowest frame rate. The algorithm has the most time to process the images and create the map. The quality declines continuously, and with 4 fps is no longer acceptable as a good quality tracking.

### 3.9. 3D Map Creation from the Map Points

As part of the goals of this work, we tried to create a 3D Map from the map points which the algorithms yield. We used the MeshLab software (http://www.meshlab.net/, 27 September 2019) for the 3D visualization. For both algorithms the results of their best version were used.

**Figure 10:**
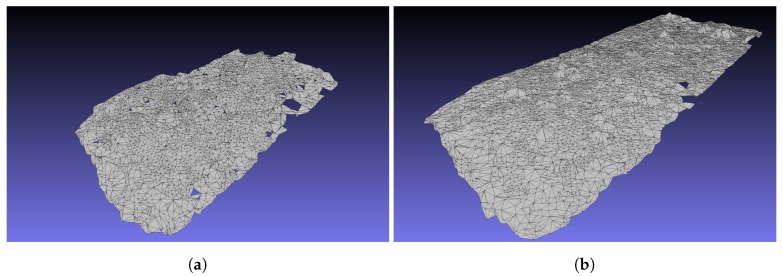
Comparison of the 3D maps of ORB- and GPS-SLAM. (**a**) 3D map of ORB-SLAM with 2000 features. (**b**) 3D map of GPS-SLAM with 6000 features.

The map points are saved into a *.txt* file by the algorithm. This file has to be converted to an *.xyz* file so that it can be imported to MeshLab. In MeshLab in order to make a surface model, the normal vectors are calculated for a neighbourhood of 10 points. After this the *Ball Pivoting* Surface Reconstruction is applied to create the surfaces. The *Ball Pivoting* method deals very well with false map points, and no after editing is needed to get a good quality 3D map. In Figure 10 the results of the 3D map reconstruction can be seen.

In the given examples. the 3D map of ORB-SLAM (see in Figure 10a) has 25,553 vertices and 15,414 faces, while the 3D map of GPS-SLAM (see in Figure 10b) has 109,116 vertices and 35,743 faces.

Since GPS-SLAM could track the path longer, a bigger map can be created from its map points. However, the map is not only bigger, but it is also denser: the map of GPS-SLAM is approximately twice as big as the one of ORB-SLAM, but it has around 4 times as many vertices. For both versions, it is true that they do not create false map points in a significant number and so their maps do not contain clearly erroneous elements.

We can declare that, not only was the quality of the tracking improved by the augmented algorithm, but a better quality 3D map can also be created from the modified algorithm’s map points.

As a comparison we reconstructed the 3D map with OpenDroneMap (https://www.opendronemap.org/, 27 September 2019). The software used only the existing images to create the 3D map. The results were of very high quality, but the execution time exceeded more than 7 h. We have a trade-off in this case. With GPS-SLAM, after tracking the path the 3D map can be reconstructed in less then a minute with good quality. Meanwhile, OpenDroneMap needs a lot of time, but yields a 3D map with very high quality. For fast solutions, GPS-SLAM is more suitable, but if the quality is very important other methods should be used.

**Figure 11:**
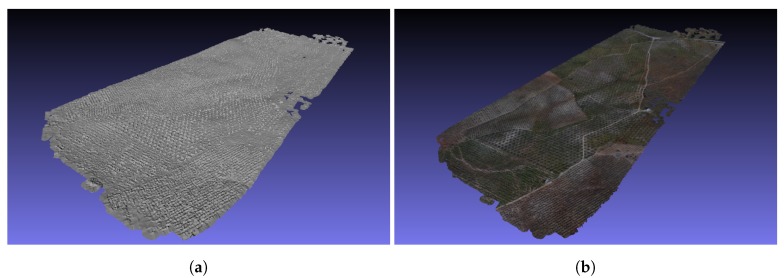
High quality 3D map reconstruction using OpenDroneMap. (**a**) 3D mesh model. (**b**) 3D texture model.

Figure 11 shows the 3D reconstructions made with OpenDroneMap.

## 4. Conclusions

Summarizing the results, we can state that the goals of the augmentation of the ORB-SLAM algorithm were reached. Starting from a partly successful tracking of the olive tree dataset by the original version, we successfully changed the functionalities of the code in a way that the tracking by GPS-SLAM became more robust and of better quality. We can declare that the additional information of the GPS and inertial data improved the algorithm.

The goal was to track a scarce dataset in a good quality in which the overlapping of the images was much smaller than in other datasets with a high frame rate. This objective was reached with good results, which were detailed in Section 3.

Processing times of the images are slightly higher for the augmented version, but they remain under the available time and so the algorithm remains real-time. There may be further improvements on the algorithm, above all regarding the relocalization method, but for the given problem, and for low frame rate datasets GPS-SLAM is capable of real-time tracking with a high quality.

## Figures and Tables

**Table 1:** The average of the extracted data from 10 runs of each algorithm made with 2000 features.

Aspects of Comparison	ORB-SLAM	GPS-SLAM	Imp.	In %	*p*-Value
Last Tracked Frame	750.67	768.6	**✓**	+2.39%	7.8 $\times \;10^{-21}$
Lost (times)	7.08	12.3	**✗**	−73.7%	0.0666
Number of Lost Frames	280	76.2	**✓**	+72.8%	9.6 $\times \;10^{-11}$
Lost Frames per Losses	39.55	6.20	**✓**	+84.3%	2.2 $\times \;10^{-9}$
N. of Calls to *TrackReferenceKeyFrame*	31.75	20.4	**✓**	+35.7%	0.0392
Number of Erroneous Frames	11.67	8.9	**✓**	+23.7%	0.31
Number of Map Points	30,391	82,614	**✓**	+172%	2.07 $\times \;10^{-4}$
Percentage of False Map Points	1.45%	7.84%	**✗**	−441%	0.0431
Mean Tracking Time [s]	0.03311	0.06020	**✗**	−81.8%	1.51 $\times \;10^{-9}$
StdDev of Tracking Time [s]	0.01152	0.02921	**✗**	−154%	2.08 $\times \;10^{-4}$
Median of Tracking Time [s]	0.03103	0.05514	**✗**	−77.7%	9.6 $\times \;10^{-20}$
Mean Relocalization Time [s]	0.01489	0.07657	**✗**	−414%	3.82 $\times \;10^{-5}$
**2000 features extracted**					

**Table 2:** The average of the extracted data from 10 runs of each algorithm made with 6000 features.

Aspects of Comparison	ORB-SLAM	GPS-SLAM	Imp.	In %	*p*-Value
Last Tracked Frame	754.92	769	**✓**	+1.87%	5.88 $\times \;10^{-3}$ notation.
Lost (times)	10	6.7	**✓**	+33%	1.01 $\times \;10^{-3}$
Number of Lost Frames	172.67	44.4	**✓**	+74.3%	1.73 $\times \;10^{-8}$
Lost Frames per Losses	17.27	6.63	**✓**	+61.6%	1.86 $\times \;10^{-6}$
N. of Calls to *TrackReferenceKeyFrame*	44.5	13	**✓**	+70.8%	8.5 $\times \;10^{-13}$
Number of Erroneous Frames	69.5	2.4	**✓**	+96.5%	7.39 $\times \;10^{-8}$
Number of Map Points	79,127	109,087	**✓**	+37.8%	8.95 $\times \;10^{-4}$
Percentage of False Map Points	8.60%	0.72%	**✓**	+91.6%	7.06 $\times \;10^{-7}$
Mean Tracking Time [s]	0.06270	0.09030	**✗**	−44.0%	1.0 $\times \;10^{-13}$
StdDev of Tracking Time [s]	0.03285	0.05320	**✗**	−61.9%	3.28 $\times \;10^{-4}$
Median of Tracking Time [s]	0.05753	0.08094	**✗**	−40.7%	9.9 $\times \;10^{-14}$
Mean Relocalization Time [s]	0.03858	0.09523	**✗**	−147%	1.73 $\times \;10^{-6}$
**6000 features extracted**					

**Table 3:** Comparison table for the ORB-SLAM executions for 2000 and 6000. Average values for 10 executions.

Aspects of Comparison	ORB 2000	ORB 6000	Improvement
Last Tracked Frame	750.67	754.92	**✓**
Lost (times)	7.08	10	**✗**
Number of Lost Frames	280	172.67	**✓**
Lost Frames per Losses	39.60	17.27	**✗**
N. of Calls to *TrackReferenceKeyFrame*	31.75	44.5	**✗**
Number of Erroneous Frames	11.67	69.5	**✗**
Number of Map Points	30,391	79,126.9	**✓**
Percentage of False Map Points	1.45%	8.60%	**✗**
Mean Tracking Time [s]	0.03311	0.06270	**✗**
StdDev of Tracking Time [s]	0.01152	0.03285	**✗**
Median of Tracking Time [s]	0.03103	0.05753	**✗**
Mean Relocalization Time [s]	0.01489	0.03858	**✗**

**Table 4:** Comparison table for the GPS-SLAM executions for 2000 and 6000. Average values for 10 executions.

Aspects of Comparison	GPS 2000	GPS 6000	Improvement
Last Tracked Frame	768.6	769	**✓**
Lost (times)	12.3	6.7	**✓**
Number of Lost Frames	76.2	44.4	**✓**
Lost Frames per Losses	6.20	6.63	**✗**
N. of Calls to *TrackReferenceKeyFrame*	20.4	13	**✓**
Number of Erroneous Frames	8.9	2.4	**✓**
Number of Map Points	82,614	109,087	**✓**
Percentage of False Map Points	7.84%	0.72%	**✓**
Mean Tracking Time [s]	0.06020	0.09027	**✗**
StdDev of Tracking Time [s]	0.02921	0.05319	**✗**
Median of Tracking Time [s]	0.05514	0.08094	**✗**
Mean Relocalization Time [s]	0.07657	0.09523	**✗**

**Table 5:** Comparison table for the best adjustments for both algorithms.

Aspects of Comparison	ORB 2000	GPS 6000	Improvement
Last Tracked Frame	750.67	769	**✓**
Lost (times)	7.08	6.7	**✓**
Number of Lost Frames	280	44.4	**✓**
Lost Frames per Losses	39.55	6.63	**✓**
N. of Calls to *TrackReferenceKeyFrame*	31.75	13	**✓**
Number of Erroneous Frames	11.67	2.4	**✓**
Number of Map Points	30,391	109,087	**✓**
Percentage of False Map Points	1.45%	0.72%	**✓**
Mean Tracking Time [s]	0.03311	0.09027	**✗**
StdDev of Tracking Time [s]	0.01152	0.05319	**✗**
Median of Tracking Time [s]	0.03103	0.08094	**✗**
Mean Relocalization Time [s]	0.01489	0.09523	**✗**
**Final comparison for the best working versions**			

## Abbreviations

The following abbreviations are used in this manuscript:

GPS	Global Positioning System
SLAM	Simultaneous Localization and Mapping
fps	frames per second
IMU	Inertial Measurement Unit
UAV	Unmanned Aerial Vehicle
GNSS	Global Navigation Satellite System

## Appendix A

### Appendix A.1. The Calculation of the Rotation Matrix between the Coordinate Systems of SLAM and GPS in Tracking.cc in Method CreateInitialMapMonocular

cv::Mat slamPosition = mCurrentFrame.mTcw.rowRange(0,3).colRange(0,3) *

mCurrentFrame.mTcw.rowRange(0,3).col(3);

cv::Mat gpsPosition = mCurrentFrame.mTGPS.rowRange(0,3).col(3) -

mFirstFrame.mTGPS.rowRange(0,3).col(3);

cout << "SLAM matrix of initializing frame: " << endl << mCurrentFrame.mTcw << endl;

cv::Mat slamPositionNorm = slamPosition/cv::norm(slamPosition);

cv::Mat gpsPositionNorm = gpsPosition/cv::norm(gpsPosition);

cv::Mat rotationAxis = crossProduct(gpsPositionNorm,slamPositionNorm) /

cv::norm(crossProduct(gpsPositionNorm, slamPositionNorm));

cv::Mat angleOfRot = cv::Mat(1,1,CV32F);

if(dotProduct(gpsPositionNorm, slamPositionNorm)<1.0){

  angleOfRot.at<float>(0) = acos(dotProduct(gpsPositionNorm, slamPositionNorm));

} else{

  angleOfRot.at<float>(0) = acos(1.0);

}

cv::Mat rotationVector;

cv::vconcat(rotationAxis,angleOfRot,rotationVector);

cout << "rotationVector: " << rotationVector << endl;

float sinOfRot = sin(rotationVector.at<float>(3));

float cosOfRot = cos(rotationVector.at<float>(3));

float tOfRot = 1-cosOfRot;

cv::Mat normRotAxis = rotationAxis/cv::norm(rotationAxis);

float xOfNormRotAxis = normRotAxis.at<float>(0);

float yOfNormRotAxis = normRotAxis.at<float>(1);

float zOfNormRotAxis = normRotAxis.at<float>(2);

mRotationOfCS.at<float>(0,0) = tOfRot*xOfNormRotAxis*xOfNormRotAxis + cosOfRot;

mRotationOfCS.at<float>(0,1) = tOfRot*xOfNormRotAxis*yOfNormRotAxis -

sinOfRot*zOfNormRotAxis;

mRotationOfCS.at<float>(0,2) = tOfRot*xOfNormRotAxis*zOfNormRotAxis +

sinOfRot*yOfNormRotAxis;

mRotationOfCS.at<float>(1,0) = tOfRot*xOfNormRotAxis*yOfNormRotAxis +

sinOfRot*zOfNormRotAxis;

mRotationOfCS.at<float>(1,1) = tOfRot*yOfNormRotAxis*yOfNormRotAxis + cosOfRot;

mRotationOfCS.at<float>(1,2) = tOfRot*yOfNormRotAxis*zOfNormRotAxis -

sinOfRot*xOfNormRotAxis;

mRotationOfCS.at<float>(2,0) = tOfRot*xOfNormRotAxis*zOfNormRotAxis -

sinOfRot*yOfNormRotAxis;

mRotationOfCS.at<float>(2,1) = tOfRot*yOfNormRotAxis*zOfNormRotAxis +

sinOfRot*xOfNormRotAxis;

mRotationOfCS.at<float>(2,2) = tOfRot*zOfNormRotAxis*zOfNormRotAxis + cosOfRot;

float ratioOfCS = cv::norm(gpsPosition)/cv::norm(slamPosition);

cout << "ratioOfCS: " << ratioOfCS << endl;

cout << "Rotation matrix, CALCULATED: " << endl << mRotationOfCS << endl << endl;

mRotationOfCS = mRotationOfCS/ratioOfCS;

cout << "Rotation matrix, DIVIDED: " << endl << mRotationOfCS << endl << endl;

### Appendix A.2. GPS Data: Calculating the Parts of the Transformation Matrix etween Two Frames in Tracking.cc in Method Track

   The rotation part:

mCurrentFrame.mtransGPS.rowRange(0,3).colRange(0,3) =
  mCurrentFrame.mT_GPS.rowRange(0,3).colRange(0,3) *
    (mLastFrame.mT_GPS.rowRange(0,3).colRange(0,3)).inv();

The translation part:

mCurrentFrame.mtransGPS.rowRange(0,3).col(3) =
  mCurrentFrame.mT_GPS.rowRange(0,3).col(3) -
    mLastFrame.mT_GPS.rowRange(0,3).col(3);

### Appendix A.3. Determination of the next camera pose in Tracking.cc in method TrackWithGPSData (Line 1242)

cv::Mat translationSLAM = mRotationOfCS * mCurrentFrame.mtransGPS.rowRange(0,3).col(3);

cv::Mat newPose = cv::Mat::eye(4,4,CV32F);

cv::Mat newRot = cv::Mat::eye(3,3,CV32F);

cv::Mat newPos = cv::Mat(3,1,CV32F);

newRot = (mCurrentFrame.mtransGPS.rowRange(0,3).colRange(0,3)) *

mLastFrame.mTcw.rowRange(0,3).colRange(0,3);

newRot.copyTo(newPose.rowRange(0,3).colRange(0,3));

newPos = (mCurrentFrame.mtransGPS.rowRange(0,3).colRange(0,3)) *

(mLastFrame.mTcw.rowRange(0,3).col(3) + (mLastFrame.mTcw.rowRange(0,3).colRange(0,3)).

inv() * translationSLAM);

newPos.copyTo(newPose.rowRange(0,3).col(3));
